# THE COURSE OF DEPRESSIVE SYMPTOMS IN OLDER ADULTS RECEIVING IN-HOME CARE

**DOI:** 10.1093/geroni/igz038.1003

**Published:** 2019-11-08

**Authors:** Anne-Sofie Helvik, maria Barca, Sverre Bergh, Jūratė Šaltytė –Benth, Tom Borza

**Affiliations:** 1 Norwegian National Advisory Unit for Aging and Health, Trondheim, Norway; 2 Norwegian National Advisory Unit on aging and health, Tønsberg, Norway; 3 Innlandet Hospital Trust, Ottestad, Norway; 4 University of Oslo, Oslo, Norway

## Abstract

The aim of the study was to describe the prevalence, incidence and persistence of depressive symptoms over a 36-month follow-up period among older people receiving in-home care, and to explore the association between cognitive function and the course of depressive symptoms. In all, 1001 older people (≥ 70 years) receiving in-home care were included in a longitudinal study over 36 months. Depressive symptoms, cognitive function, general medical health, activities of daily living, neuropsychiatric symptoms and use psychotropic drugs were assessed at three assessments. Dementia and mild cognitive impairment were diagnosed at all assessments. Baseline demographic characteristics and information on nursing home residency at follow-up were recorded. Linear mixed models were estimated. We found the prevalence and cumulative incidence of individual depressive symptoms to be higher in those with dementia at baseline than in those without. The persistence of depressive symptoms did not differ between those with or without dementia at baseline. The severity of cognitive decline and mean depressive symptom score assessed simultaneously were positively associated, but the strength of the association changed over time and was not significant at the last assessment. In conclusion: The differences in prevalence and cumulative incidence of depressive symptoms in those with and without dementia at baseline, and the association found between degree of cognitive decline and depressive symptoms over time shows that depression and dementia are interconnected. Nurses and clinicians should pay attention to cognitive status when observing or evaluating depression among older people receiving in-home care.

